# Supplementary material for: Modeling human HSV infection via a vascularized immune-competent skin-on-chip platform
Source: Nat Commun. 2022 Sep 19;13:5481. doi: 10.1038/s41467-022-33114-1 (PMC9485166; doi:10.1038/s41467-022-33114-1)
Supplement: Supplementary file 1 — Supplementary Information [file 41467_2022_33114_MOESM1_ESM.pdf]

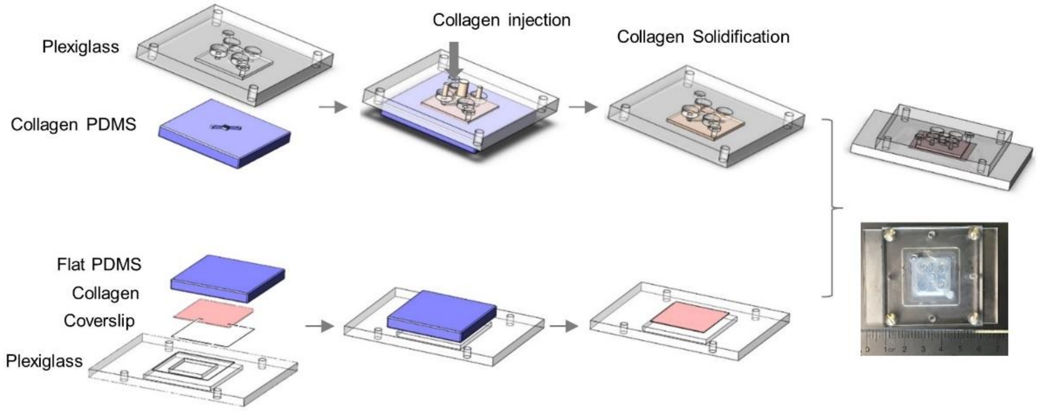

Supplemental Figure 1. Schematic fabrication of the microfluidic network in the fibroblast containing collagen. A PDMS stamp with channel geometry was placed at the bottom of the top plexiglass jig and fibroblast-containing collagen was injected through the inject ports into the top well. Two dowel pins were inserted into perfusion ports to define the space for inlet and outlet, and a flat piece of stainless steel was placed on top of the open well to create a flat collagen surface for keratinocyte culture. The bottom plexiglass also provided a well for collagen. After fibroblast-containing collagen was added into the well, a flat PDMS stamp was placed on top to create a flat collagen gel. Collagen in both top and bottom plexiglass was incubated at 37°C for 25 minutes for gelation. PDMS stamps were subsequently removed, and top and bottom plexiglass jigs were assembled and sealed by mechanical pressure.

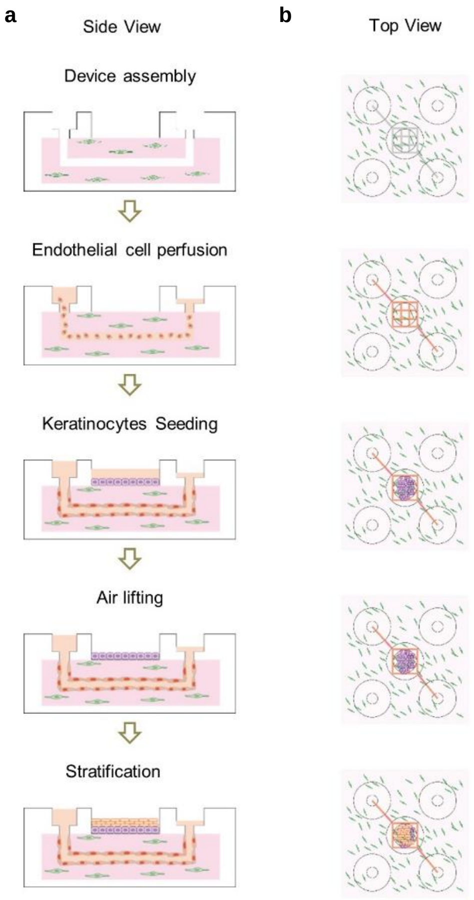

Supplemental Figure 2. Schematic description of the protocol to develop skin-on-a-chip. **a** Cross-sectional and **b** horizontal views of the skin-on-chip device during various steps of production. After microfluidic channel was created inside the fibroblast-containing collagen within a plexiglass jig, endothelial cells were then perfused through the microfluidic channel. Endothelial cells attached to the channel wall and formed vasculature and keratinocytes were added to the middle well. Once the keratinocytes reached confluency, keratinocytes were kept in differentiation medium for one additional day. The medium was then removed, forming an air-liquid interface, which allowed for keratinocyte differentiation and multilayer epidermis formation.

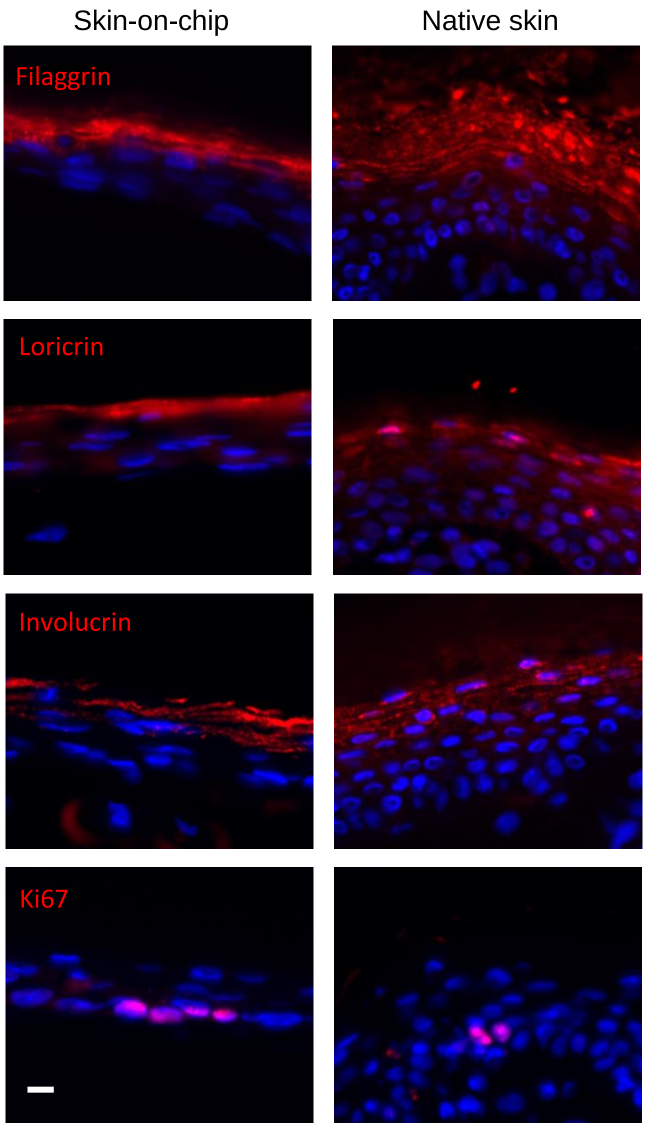

Supplemental Figure 3. Expression of keratinocyte differentiation markers in skin-on-chip and native human skin. Expression of filaggrin, Loricrin, Involucrin and Ki67 expression in stratified epidermis in the skin-on-chip (left), and in a representative normal human skin biopsy (right); blue: DAPI. Scale bar, 20 µm.

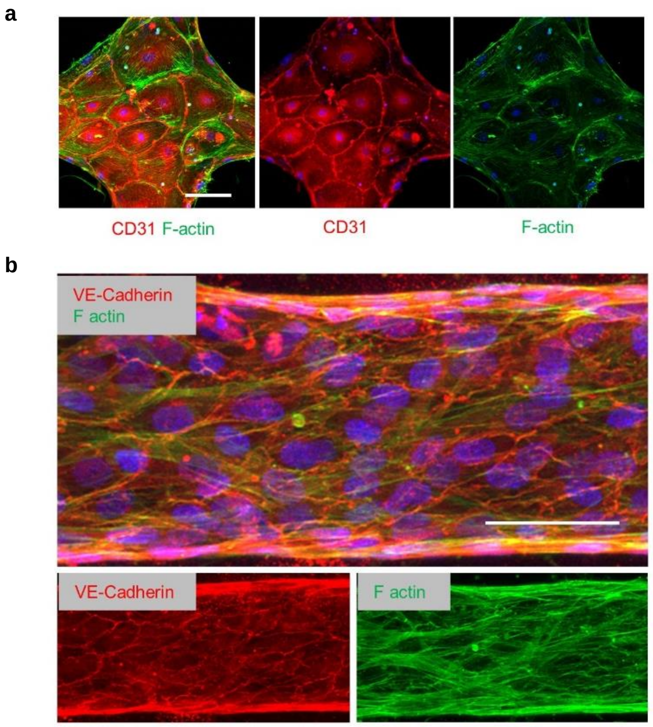

Supplemental Figure 4. Expression of cellular junction proteins on endothelial cells in skin-on-chip. a CD31 and **b** VE-Cadherin expression. Red: CD31 or VE-Cadherin; Green: F-actin; Blue: DAPI. Scale bar:100µm.

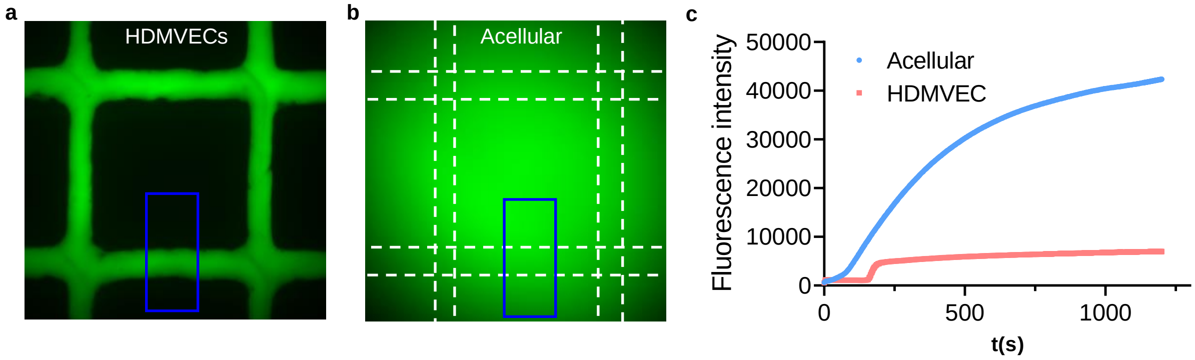

Supplemental Figure 5. FITC-Dextran perfusion of skin-on-chip. Fluorescence images of FITC-Dextran-perfused vessels with **a** or without **b** human dermal microvascular endothelial cells (HDMVECs). Dash lines indicated microvascular channel walls in device without HDMVECs. **c** Kinetics of FITC-Dextran diffusion in the skin-on-chip with or without HDMVECs. Monitoring fluorescence intensity of boxed area for 1200 seconds (20 mins) from the start of perfusion. Source data are provided as a Source Data file.

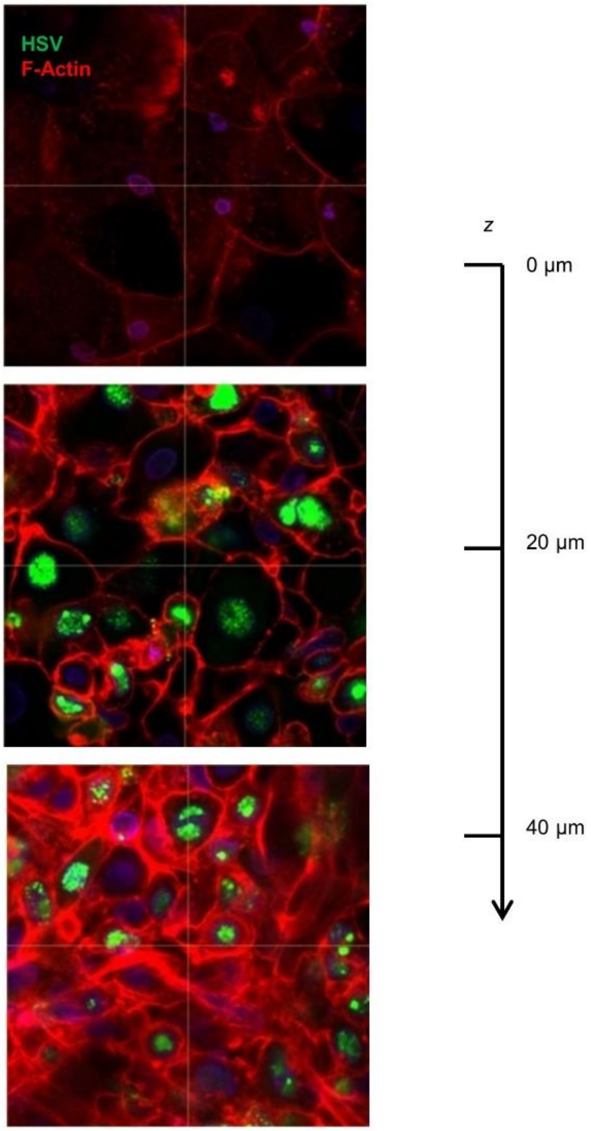

Supplemental Figure 6. HSV infection in the stratified layers of epidermis. Mechanically disrupted epidermis infected by HSV is shown from superficial (top) to basal (bottom) keratinocytes. Green: HSV-1 strain K26; Red: F-actin; Blue: DAPI.

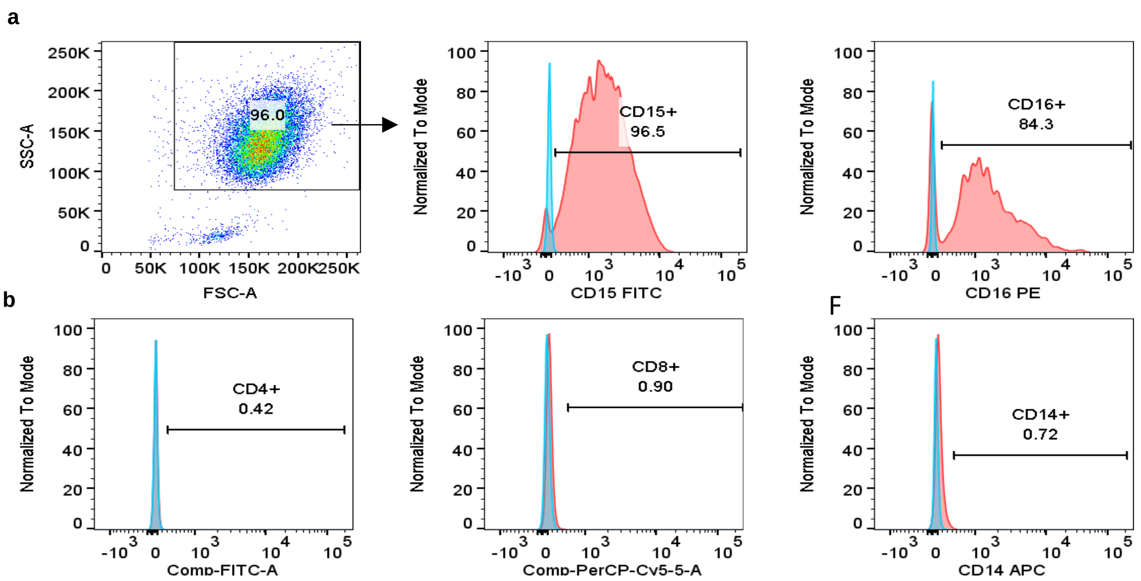

Supplemental Figure 7. Flow cytometry analysis of purified neutrophils. a Flow plots showing neutrophil marker expression (CD15 and CD16) on the purified cell population following neutrophil isolation protocol. **b** Expression of CD4 and CD8 T-cell markers and CD14 monocytes/macrophage marker are minimum in the purified neutrophil population. Live cells gated using SSC x FSC. Red histograms: stained cells, Blue histograms: unstained cells.

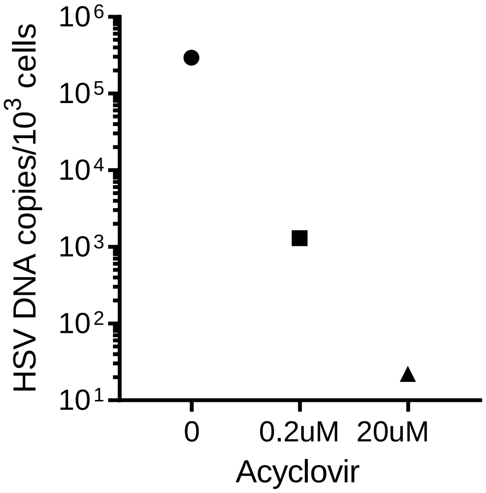

Supplemental Figure 8. HSV viral replication following treatment with Acyclovir. HSV genome copy numbers quantified by real-time qPCR in skin-on-chip treated with increasing concentrations of acyclovir. n=1-2 for each condition. Source data are provided as a Source Data file.
