# Supplementary material for: Modeling human HSV infection via a vascularized immune-competent skin-on-chip platform
Source: Nat Commun. 2022 Sep 19;13:5481. doi: 10.1038/s41467-022-33114-1 (PMC9485166; doi:10.1038/s41467-022-33114-1)
Supplement: Supplementary file 6 — Reporting Summary [file 41467_2022_33114_MOESM6_ESM.pdf]

nature research

Corresponding author(s): Jia Zhu, NCOMMS-20-02891-B

Last updated by author(s): Sep 1, 2022

Reporting Summary

Nature Research wishes to improve the reproducibility of the work that we publish. This form provides structure for consistency and transparency in reporting. For further information on Nature Research policies, see Authors & Referees and the Editorial Policy Checklist .

Statistics

For all statistical analyses, confirm that the following items are present in the figure legend, table legend, main text, or Methods section.

n/a	Confirmed	
[ ]	[x]	The exact sample size (*n*) for each experimental group/condition, given as a discrete number and unit of measurement
[ ]	[x]	A statement on whether measurements were taken from distinct samples or whether the same sample was measured repeatedly
[ ]	[x]	The statistical test(s) used AND whether they are one- or two-sided<br>*Only common tests should be described solely by name; describe more complex techniques in the Methods section.*
[ ]	[x]	A description of all covariates tested
[x]	[ ]	A description of any assumptions or corrections, such as tests of normality and adjustment for multiple comparisons
[ ]	[x]	A full description of the statistical parameters including central tendency (e.g. means) or other basic estimates (e.g. regression coefficient) AND variation (e.g. standard deviation) or associated estimates of uncertainty (e.g. confidence intervals)
[ ]	[x]	For null hypothesis testing, the test statistic (e.g. *F*, *t*, *r*) with confidence intervals, effect sizes, degrees of freedom and *P* value noted<br>*Give P values as exact values whenever suitable.*
[x]	[ ]	For Bayesian analysis, information on the choice of priors and Markov chain Monte Carlo settings
[x]	[ ]	For hierarchical and complex designs, identification of the appropriate level for tests and full reporting of outcomes
[x]	[ ]	Estimates of effect sizes (e.g. Cohen's *d*, Pearson's *r*), indicating how they were calculated

Our web collection on statistics for biologists contains articles on many of the points above.

Software and code

Policy information about availability of computer code

Data collection	Live cell images: high resolution Photometrics Coolsnap HQ2 scientific CCD camera equipped on a Nikon Ti microscope<br>Cytokines: measured on a Luminex 200 instrument<br>Fixed tissues: imaged on Nikon Ti microscope equipped with a CCD camera<br>Flow cytometry: BD Canto II
Data analysis	FACSDiva (version 8.0, BD Biosciences)<br>Prism (version 9.0.2, GraphPad)<br>ImageJ (version 2.3.0/1.53q, NIH)<br>Volocity (version 6.5.0, Quorum Technologies)<br>FlowJo (version 9.8.8, BD Biosciences)<br>Solidworks (version SP3)

For manuscripts utilizing custom algorithms or software that are central to the research but not yet described in published literature, software must be made available to editors/reviewers. We strongly encourage code deposition in a community repository (e.g. GitHub). See the Nature Research guidelines for submitting code & software for further information.

Data

Policy information about availability of data

All manuscripts must include a data availability statement. This statement should provide the following information, where applicable:

- Accession codes, unique identifiers, or web links for publicly available datasets
- A list of figures that have associated raw data
- A description of any restrictions on data availability

Source data are provided with this paper for figures with associated raw data: 1, 2, 4, 5, 6, 7, S4, S8.

Field-specific reporting

Please select the one below that is the best fit for your research. If you are not sure, read the appropriate sections before making your selection.

- [x] Life sciences
- [ ] Behavioural & social sciences
- [ ] Ecological, evolutionary & environmental sciences

For a reference copy of the document with all sections, see nature.com/documents/nr-reporting-summary-flat.pdf

Life sciences study design

All studies must disclose on these points even when the disclosure is negative.

Sample size	We followed the statistical principles in hypothesis testing with two-sample inference in estimation of sample size and power for comparison (Rosner B., Fundamentals of Biostatistics, 7th Edition, 2010). Based on the pilot study and our previous experimental experience, all in vitro experiments relating to HSV infection and ACV drug treatments were performed with independent biological replicates for each condition indicated in the figure legend, which has a calculated test power of >0.998 for assessing significance level at 0.05.
Data exclusions	No data was excluded.
Replication	All experiments were independently performed and replicated, as indicated in the figure legends, to ensure reproducibility of the results.
Randomization	Skin on chip constructs were randomly assigned for mock or treatment condition (HSV infection, antiviral, or antibody).
Blinding	The experiments measuring cytokines and chemokines were performed in a blinded manner by the Immune Monitoring Core at Fred Hutchinson Cancer Center. For experiments comparing ACV drug perfusion dosage (e.g. 0, 0.2 and 20 uM) and time (e.g. 24hr prior vs 0hr vs 24hr post infection), blinding was not applied because experimental conditions needed to be determined prior infection of HSV-1 or HSV-2 strains. In these experiments, we measured the entire epidermis for viral infection level in individual cells of all samples and conditions tested.

Reporting for specific materials, systems and methods

We require information from authors about some types of materials, experimental systems and methods used in many studies. Here, indicate whether each material, system or method listed is relevant to your study. If you are not sure if a list item applies to your research, read the appropriate section before selecting a response.

Materials & experimental systems

n/a	Involved in the study	
☐	☒	Antibodies
☐	☒	Eukaryotic cell lines
☒	☐	Palaeontology
☒	☐	Animals and other organisms
☐	☒	Human research participants
☒	☐	Clinical data

Methods

n/a	Involved in the study	
☒	☐	ChIP-seq
☐	☒	Flow cytometry
☒	☐	MRI-based neuroimaging

Antibodies

Antibodies used

rabbit anti-human keratin 14 (1:25, Abcam, ab192056)
rabbit anti-human keratin 10 (1:25, Abcam, ab194229)
mouse anti-type IV collagen (1:100, Sigma-Aldrich, C1926)
rabbit anti-human vimentin (1:100, Abcam ab92547)
mouse anti-human filaggrin (1:50, Invitrogen, MA5-13440)
rabbit anti-human loricrin (1:50, Abcam, ab85679)
mouse anti-human involucrin (1:50, Invitrogen, MA5-11803)
rabbit anti-human Ki67 (1:50, Abcam, ab15580)
rabbit anti-human CD31(1:50, Abcam, ab32457)
mouse anti-human CD15 (1:50, BD Biosciences, 555400)
rabbit anti-human VE-cadherin (1:50, Abcam, ab33168)
rabbit anti-human HSV-2 (1:500, Dako, B0116)
CD4-FITC (1:100, BD Biosciences, 340133)
CD8-PerCP-Cy5.5 (1:100, BD Biosciences, 341051)
CD14-APC (1:100, BD Biosciences, 561708)
CD15-FITC (1:100, BD Biosciences, 560997)
CD16-PE (1:50, BD Biosciences, 555407)

Alexa Fluor 488 goat anti-mouse IgG antibody (1:100, Invitrogen, A11001)
Alexa Fluor 647 goat anti-rabbit IgG antibody (1:100, Invitrogen, A21244)
Alexa Fluor 594 donkey anti-mouse IgG antibody (1:100, Invitrogen, A21203)
Alexa Fluor 546 donkey anti-rabbit IgG antibody (1:100, Invitrogen, A10040)
F-actin were stained with Alexa Fluor 594 phalloidin (1:1000, Thermo Fisher Scientific, A12381)

Validation

All antibodies are commercially available and validated by the manufacturer as follows.
Abcam: Antibody specificity is confirmed by looking at cells that either do or do not express the target protein within the same tissue. Initially, our scientists will review the available literature to determine the best cell lines and tissues to use for validation. We then check the protein expression by IHC/ICC to see if it has the expected cellular localization (Figure 3). If the localization of the signal is as expected, this antibody will pass and is considered suitable for use in IHC/ICC.
Sigma-Aldrich: Delivering high-quality antibodies requires us to perform rigorous specificity and sensitivity testing in order to provide our customers with reliable tools that generate consistent results. We routinely perform standard validation processes across our antibody portfolio. Our standard antibody validation processes include verification for each recommended immunodetection application. Each of the thousands of antibodies in our portfolio are certified through our standard validation process to ensure quality and reproducibility.
Invitrogen/Thermo Fisher: antibodies are currently undergoing a rigorous two-part testing approach. 1) Target specificity verification. This helps ensure the antibody will bind to the correct target. Our antibodies are being tested using at least one of the following methods to ensure proper functionality in researcher's experiments. 2) Functional application validation. These tests help ensure the antibody works in a particular application(s) of interest.
BD: antibody specificity is confirmed using multiple methodologies that may include a combination of flow cytometry, immunofluorescence, immunohistochemistry or western blot to test staining on a combination of primary cells, cell lines or transfectant models.
Dako: The robust IHC tests are calibrated and validated for reliable diagnostic use, ensuring that the antigen is correctly demonstrated at both high and low expression levels in tissue to support your lab in reducing the risk of false negative and false positive results.

Eukaryotic cell lines

Policy information about cell lines

Cell line source(s)	Vero cells (ATCC, cat# CCL-81)
Authentication	Authenticated based on morphology
Mycoplasma contamination	Cell lines tested negative
Commonly misidentified lines (See ICLAC register)	No commonly misidentified lines were used

Human research participants

Policy information about studies involving human research participants

Population characteristics

Study participants are HSV-2 seropositive and HIV-1 seronegative healthy individuals (n=20), who has a history of culture proven recurrent genital HSV-2 disease, with no upper age limit. Participants include both male (n=4) and female (n=16), between 20-71 years old. Minority representation reflects the local population in Seattle, Washington, US.

Recruitment

The skin biopsy tissue were obtained from University of Washington Virology Research Clinic (UW-VRC), where a cohort of HSV-2 seropositive persons ≥18 years old were recruited. Eligible participants, both male and female, with genital HSV-2 outbreaks in sites amenable to biopsy as defined in our IRB approved protocol were included. The participants were compensated fifty dollars for each biopsy they donated. We excluded persons who were HIV seropositive, had a history of poor wound healing or had known allergies to local anesthesia. Pregnant women were also excluded. Biopsies were performed only in areas which are unlikely to cause sexual or functional compromise. Clitoral/periurethral areas in women and penile shaft and scrotum in men are to be avoided. We realize that participants are likely those with intermittent or frequent HSV-2 recurrence. People with asymptomatic HSV reactivation are not included in our tissue-based studies. Thus, results drawn from our study might only characterize human clinical symptomatic HSV-2 reactivation.

The UW-VRC has conducted clinic studies, which have defined our understanding of the natural history, pathogenesis, and immunology of herpes simplex virus infections for almost fifty years. Advertisements for the study were posted on the clinical website, news letters and in local newspapers throughout the Seattle area. This might result in self-selecting for individuals who read those news sources and are local to the area. Our clinics have longstanding experience in participant recruitment and aim to make the cohort reflective of the age and racial demographics of the general population in the greater Seattle area.

Ethics oversight

The study protocol involving human specimens was approved by the University of Washington Institutional Review Board Committee (STUDY00002443), and written informed consents were obtained from all participants including consent to publish.

Note that full information on the approval of the study protocol must also be provided in the manuscript.

Flow Cytometry

Plots

Confirm that:

- [x] The axis labels state the marker and fluorochrome used (e.g. CD4-FITC).
- [x] The axis scales are clearly visible. Include numbers along axes only for bottom left plot of group (a 'group' is an analysis of identical markers).
- [x] All plots are contour plots with outliers or pseudocolor plots.
- [x] A numerical value for number of cells or percentage (with statistics) is provided.

Methodology

Sample preparation	Neutrophils were isolated from fresh human whole blood. Briefly, whole blood from a healthy donor was collected in an acid citrate dextrose tube. After dilution with HBSS (no calcium, no magnesium) (Gibco), the blood mixture was layered onto Ficoll-Paque Premium (GE healthcare life sciences) and centrifuged at 850 g for 20 mins with brake off. The neutrophil- and erythrocyte-rich layer at the bottom was mixed with 3% Dextran (MP Biomedicals) and sat for 20 mins at room temperature allowing erythrocytes to sediment under gravity. The neutrophil-rich supernatant was transferred into a fresh tube and centrifuged at 300 g for 10 mins. The cell pellet was incubated with ACK lysing buffer (Gibco) for 5 mins to lyse the remaining erythrocytes. The cells were then washed with HBSS twice and resuspended in RPMI 1640 with 10% fetal bovine serum and 1X penicillin streptomycin (Gibco).
Instrument	BD Canto II
Software	FACSDiva and FlowJo
Cell population abundance	Cell sorting was not performed
Gating strategy	FSC-A/SSC-A was used to remove debris. Expression for each marker was determined based on an unstained control.

- [x] Tick this box to confirm that a figure exemplifying the gating strategy is provided in the Supplementary Information.
