# Supplementary material for: Modeling human HSV infection via a vascularized immune-competent skin-on-chip platform
Source: Nat Commun. 2022 Sep 19;13:5481. doi: 10.1038/s41467-022-33114-1 (PMC9485166; doi:10.1038/s41467-022-33114-1)
Supplement: Supplementary file 8 — Description of Additional Supplementary Files [file 41467_2022_33114_MOESM8_ESM.pdf]

Title: Supplementary Data 1.

Description: Primary antibody summary.

Title: Supplementary Movie 1.

Description: FITC-Dextran (green) perfusion of microvascular network in a skin-on-chip coated with human dermal microvascular endothelial cells.

Title: Supplementary Movie 2.

Description: FITC-Dextran (green) perfusion of microvascular network in a skin-on-chip constructed without human dermal microvascular endothelial cells.

Title: Supplementary Movie 3.

Description: Neutrophil trans-endothelium extravasation and migration in response to epidermal infection of HSV-1, as shown in Figure 4d.
